# Glutaraldehyde-Induced Allergic Contact Dermatitis: A Case Report and Safety Standards

**DOI:** 10.7759/cureus.56954

**Published:** 2024-03-26

**Authors:** Pragya Pandey, Ramesh Bharti, Neha Jasrasaria

**Affiliations:** 1 Conservative Dentistry and Endodontics, King George's Medical University, Lucknow, IND

**Keywords:** occupational risk exposure, glutaraldehyde, delayed hypersensitivity, contact dermatitis, allergic reaction

## Abstract

Glutaraldehyde (GA), a potent disinfectant and sterilizing agent extensively used in healthcare settings, has garnered attention for its association with contact dermatitis. This occupational skin condition, often induced by repeated exposure to GA, poses significant challenges to the well-being of healthcare professionals and patients alike. Understanding the causes, symptoms, and preventive measures against GA-induced contact dermatitis is essential for promoting a safe and healthy working environment in healthcare facilities.

A 28-year-old female presented with a severe burning sensation and dark brown patches in the lower chin region, one day following root canal treatment. Based on the characteristic appearance of patches and the typical burning sensation associated with an allergic reaction, a diagnosis of acute contact dermatitis was made. Patch testing by an expert dermatologist confirmed that the patient was allergic to GA. GA, a popular commercial germicidal product, is widely used as a cold sterilizing agent for operative dental instruments. The patient developed a reaction as the endodontic files used during the root canal procedure were cold sterilized with 2% GA. The lesion experienced significant improvement and ultimately healed following the administration of corticosteroids and antihistamines. This report concerns a case of GA-induced contact dermatitis. As GA is being used more widely, particularly in dental clinics, this case was of interest and is reported in the safety interest of patients and clinicians.

## Introduction

Glutaraldehyde (GA) has been widely used as a sterilizing agent and chemical disinfectant for medical and dental equipment since the early 1960s [1]. GA is a simple, saturated five-carbon dialdehyde with the formula CHO-CH2-CH2-CH2-CHO [2]. GA offers significant advantages as a potent, rapid, and cold sterilizing agent, while also being non-corrosive and cost-effective [3,4].

The adoption of GA stemmed from the need for a safer alternative to formaldehyde [1]. Occupational Safety Health Administration (OSHA) regulated the use of formaldehyde by healthcare workers when it was listed as a potential human carcinogen [5]. Like formaldehyde, the biocidal activity of GA stems from its ability to alkylate sulfhydryl, hydroxyl, carboxyl and amino groups on organic molecules. Following alkylation, RNA, DNA, and proteins, which are the organic molecules present in micro-organisms are rapidly denatured leading to bactericidal, fungicidal, virucidal and sporicidal activity [6].

Despite its effectiveness as a sterilizing and disinfecting agent, prolonged exposure to GA has been associated with an array of adverse health effects, including occupational asthma, breathing difficulties, respiratory irritation, eye itching, rhinitis and skin rashes [3,7]. The first documented case of induced allergic contact dermatitis (ACD) in healthcare workers was by Sanderson and Cronin et al. in 1968 [8]. However, none of the reports to date have mentioned ACD in dental patients due to the use of instruments disinfected with GA.

This report presents a case of a GA-induced ACD patient undergoing a root canal treatment procedure. The article attempts to emphasize the need to improve safety standards in GA usage for the welfare of patients and healthcare professionals alike.

## Case presentation

A 28-year-old female patient reported to the Department of Conservative Dentistry and Endodontics in King George’s Medical University, Lucknow, with the chief complaint of spontaneous pain in the upper anterior tooth region from the past two days. After clinical and radiological evaluation, a diagnosis of symptomatic irreversible pulpitis was made with respect to 11 and 13 (maxillary right central incisor and maxillary right canine). Root canal treatment was recommended for the offending teeth. An undergraduate final-year student undertook the case. Medical history was non-contributory and revealed no episode of allergic response to any drug. Viral markers were investigated with blood tests. The patient was non-reactive to HIV and hepatitis B surface antigen (HBsAg). Oral prophylaxis was done before starting the treatment.

Local anaesthesia (2% lignocaine 1:200000 epinephrin) was achieved by administering an infraorbital nerve block. Isolation of the tooth was accomplished with a 5x5 rubber dam. The root canal treatment was initiated in tooth 11 with access opening using Endo access bur and EndoZ tapered safe end bur (Dentsply Maillefer, Switzerland). Negotiation of root canals was done with a size 10 K file followed by working length measurement using #15 K-File (Dentsply Mallifer). Endodontic hand files used for the cleaning and shaping of the tooth had initially been autoclaved. During the operative procedure, GA solution (Korsoster glutaraldehyde USP: 2.45% w/v)) was used for rapid chair-side disinfection for endodontic files. Apical preparation was completed to size #40 by step back technique. Ethylenediaminetetraacetic acid (EDTA) lubrication (RC-Prep, Dental Compare, USA) and constant irrigation of 3% NaOCl and saline at each change of file were done. Calcium hydroxide intracanal medicament was given and temporary cement was placed. The patient was recalled the next day for the completion of the root canal procedure.

**Figure 1:**
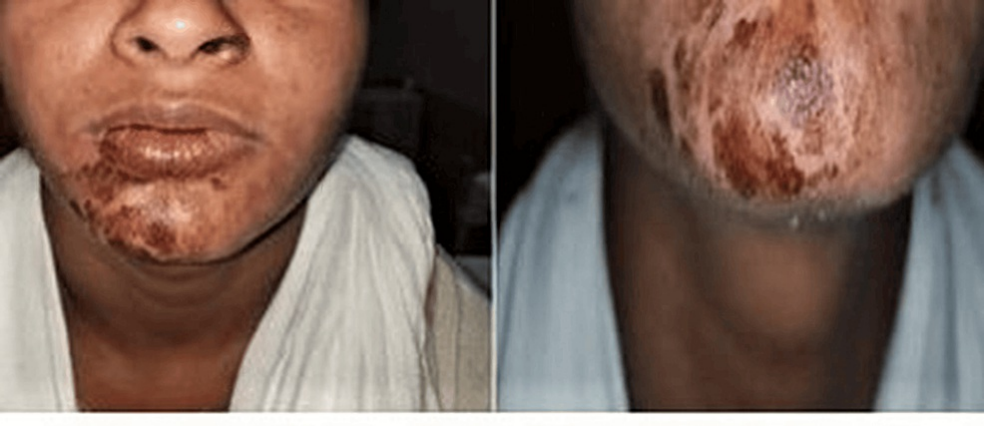
Extensive brown patches around the chin area of the patient

The patient returned the subsequent day exhibiting signs of allergic reaction, including itching and a severe burning sensation around the chin region (Figure 1). Extensive brown patches of varying sizes and shapes were seen in the affected area. A potential allergic reaction to a medication that was used during the root canal procedure was suspected. Reviewing the signs and symptoms and how the case was done, a provisional diagnosis of hypersensitivity to either GA or sodium hypochlorite was made. The patient was referred to a dermatologist for investigation to determine if the reported reaction was a reaction to GA itself or other medications administered during the procedure or any other factor. Patch testing revealed a positive response to GA and negative responses to eugenol, zinc oxide, and sodium hypochlorite.

Following the diagnosis of GA-induced ACD, the patient received comprehensive medical management aimed at alleviating symptoms and facilitating recovery. To mitigate the allergic response, the patient was reassured and initiated on a therapeutic course of Prednisolone dispersible tablet 40mg once daily for an initial period of two days, with subsequent tapering to 20mg daily over the next three days. Complementing this systemic treatment ranitidine tablets, 150mg once daily for five days, and fexofenadine hydrochloride tablet, 180mg once daily for five days, were also prescribed.

Recognizing the importance of local skin care, the patient was advised to apply Cetaphil moisturizer (Galderma Laboratories, Lausanne, Switzerland) topically twice daily to soothe irritated skin and promote hydration until symptomatic improvement was achieved. Close monitoring of the patient's progress was ensured through scheduled follow-up appointments every alternate day, allowing for timely assessment of treatment response and adjustment if necessary.

**Figure 2:**
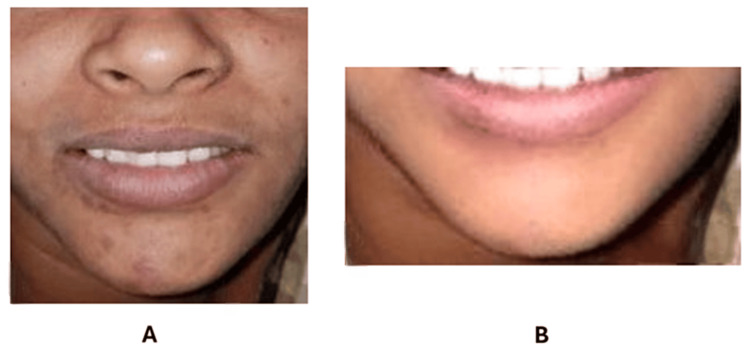
Improvement of the allergic symptoms on (A) the fifth day and (B) after three months

Over the course of treatment, a gradual amelioration of facial rashes and associated symptoms was observed, culminating in significant improvement noted by the fifth day of therapy (Figure 2A). Concurrently, the residual marks of the allergic reaction exhibited notable diminishment, with nearly complete resolution observed within a span of two weeks. No relapse or recurrence was observed during the three-month follow-up (Figure 2B).

## Discussion

The utilization of 2% GA for sterilization purposes in endodontic settings, dental operating areas, and dental impressions stands as a widely recommended practice [9]. The popularity of GA as a sterilizing agent for endodontic files and reamers is attributed to its ease of use, absence of adverse effects, and rapid sterilization capabilities. Ritchie and Gary Max tested GA as a cold sterilizing agent for endodontic instruments by leaving the endodontic files in 2% GA for a period of 14 days. They observed no visible corrosion of the metal portions, softening of the rubber stops and dulling of the cutting edges [4].

Despite its effectiveness, GA has been associated with toxic side effects, notably ACD [3,8]. The National Institute of Occupational Safety and Health has issued guidelines outlining best practices for the safe use of GA [10]. In this instance, the patient experienced contact dermatitis due to the use of instruments sterilized with GA. While most documented cases of GA-related ACD involve healthcare workers, our case highlights a unique scenario wherein patient exposure occurred through indirect contact with the sterilizing agent. Among healthcare workers, Ravis et al. found a preponderance of these allergic reactions among dental hygienists, dental assistants, and other dental personnel [11].

In the present case, the clinician wore latex gloves, this protective barrier effectively shielded their skin from direct contact with GA. However, a critical oversight occurred when the patient's chin inadvertently came into contact with GA subsequent to handling endodontic files initially sterilized with GA. Unlike many antibody-mediated allergic reactions, GA-induced dermatitis typically manifests as type IV or delayed hypersensitivity, often taking several days to develop [9]. Tammannavar et al. reported a case of immediate allergic contact urticaria to eugenol during dental treatment in which the patient developed rashes about one minute after the zinc oxide eugenol placement [12]. Our patient showed a typical presentation of ACD, which often manifests as a rash, that begins several hours after contact and like irritant dermatitis, is usually confined to the area of contact as in this case.

Studies evaluating glove permeability to 2% GA solutions showed that latex gloves exhibited breakthrough at 45 minutes, while nitrile rubber, butyl rubber, synthetic surgical gloves, and polyethene were each impermeable for at least four hours [13]. Stringent protocols recommend the use of personal protective equipment (PPE) including gloves and eye protection while handling GA. Following disinfection, the instruments should be rinsed thoroughly with sterile water. Each rinse should be a minimum of one minute in duration, and a large volume of fresh water must be used for each rinse [5,10]. However, lapses in adherence to these guidelines or insufficient awareness among healthcare workers may contribute to the escalating incidence of GA-induced contact dermatitis.

In the present case, the allergic reaction likely stemmed from residual GA on the instrument, inadvertently contacting the gloved finger of the clinician and subsequently transferring to the patient's chin when the dentist rested their fingers in that area. In a study by Nethercott et al. 10 employees who were followed for six months after initial diagnosis of ACD from GA continued to have persistent hand eczema [14].

Despite established guidelines, the rising prevalence of GA-induced contact dermatitis among healthcare workers warrants heightened attention. Potential explanations for this trend include deficiencies in guideline implementation, inadequacies in guideline effectiveness, or simply a lack of awareness among healthcare professionals.

To address these concerns, healthcare employers and employees must prioritize understanding and mitigating exposure to GA. Furthermore, the exploration of alternative methods for cold sterilization, such as chairside glass bead sterilizers, offers promising avenues for reducing reliance on GA. For those utilizing GA, comprehensive training, monitoring of personal protective measures, exposure monitoring, proper disposal practices, and adherence to spill cleanup procedures are imperative for ensuring occupational safety and minimizing adverse health outcomes.

## Conclusions

It is in the best interest of those in the healthcare profession and other professions exposed to GA to enhance occupational safety standards and to improve methods of barrier protection in the best interest of the health workers and patients. It is imperative to explore safer alternatives to GA that do not pose risks of cytotoxicity or allergic reactions. As healthcare workers, it is crucial to uphold the principle of "first do no harm" or "primum non nocere" in our practices. By prioritizing the adoption of safer alternatives and adhering to rigorous safety protocols, we can mitigate the risk of GA contact dermatitis and ensure the well-being of both patients and healthcare professionals.
